# Whole-body biodistribution of [^18^F]SMBT-1: a novel PET tracer for monoamine oxidase B imaging in healthy humans

**DOI:** 10.1007/s12149-025-02144-2

**Published:** 2026-01-07

**Authors:** Berihu Mesfin, Yui Ishioka, Yoshiki Ichinose, Akihito Inamura, Yingying Wu, Shoichi Watanuki, Kotaro Hiraoka, Yoshihito Funaki, Asuka Kikuchi, Kazuko Takeda, Masayasu Miyake, Ryuichi Harada, Shozo Furumoto, Nobuyuki Okamura, Kazuhiko Yanai, Hiroshi Watabe, Manabu Tashiro

**Affiliations:** 1 Nuclear Medicine Laboratory, Research Center for Accelerator and Radioisotope Science, Tohoku University, 6 − 3 Aramaki Aza Aoba, Aoba-ku, Sendai, Miyagi 980–8578 Japan; 2 Radiation Protection and Safety Control Laboratory, Research Center for Accelerator and Radioisotope Science, Tohoku University, Sendai, Japan; 3 Radiopharmaceutical Laboratory, Research Center for Accelerator and Radioisotope Science, Tohoku University, Sendai, Japan; 4 Department of Diagnostic Radiology, Tohoku University Hospital, Tohoku University, Sendai, Japan; 5 Division of Pharmacology, Faculty of Medicine, Tohoku Medical and Pharmaceutical University, Sendai, Japan; 6 Department of Pharmacology, Graduate School of Medicine, Tohoku University, Sendai, Japan

**Keywords:** [^18^F]SMBT-1, Biodistribution, PET, MAO-B, Neuroinflammation

## Abstract

**Objective:**

[^18^F]SMBT-1 is a selective and reversible monoamine oxidase B (MAO-B) radiotracer used for astrogliosis imaging. This study aimed to observe the whole-body biodistribution of [¹⁸F]SMBT-1 and evaluate peripheral MAO-B expression in healthy human volunteers using dynamic positron emission tomography (PET) imaging.

**Methods:**

Six healthy subjects (four males, two females; age range: 21–63 years) underwent nine dynamic PET scans over 5.5 h after [^18^F]SMBT-1 injection. The first five emission scans were acquired consecutively in a single session within 30 min (min) of post-injection (p.i.). The remaining four emission scans were performed at 70–110, 150–180, 220–250, and 290–330 min p.i. Regions of interest (ROIs) were manually drawn on the co-registered PET and magnetic resonance imaging (MRI) for the selected organs to extract mean standardized uptake values (SUV_mean_) and generate time-activity curves (TACs).

**Results:**

A significantly high early uptake of [^18^F]SMBT-1 was observed in the kidneys, liver, heart, and stomach between 5 and 30 min p.i. The kidneys showed the highest early peak at 5 min p.i. (SUV_mean_ = 14.2 ± 3.5). The gallbladder and intestines exhibited a delayed uptake pattern, with the gallbladder SUV_mean_ increasing substantially from 9.0 ± 4.2 at 30 min to 123.7 ± 53.4 at 330 min. No significant differences in tracer uptake patterns were observed across participants.

**Conclusion:**

[¹⁸F]SMBT-1 exhibited favorable reversible kinetics in the whole-body biodistribution assessment, confirming its established utility for imaging reactive astrocytes and indicating its potential for future applications in systemic high MAO-B-related pathologies.

**Supplementary Information:**

The online version contains supplementary material available at 10.1007/s12149-025-02144-2.

## Introduction

Monoamine oxidase B (MAO-B) is a flavoprotein enzyme located in the outer mitochondrial membrane [1]. It catalyzes the oxidative deamination of endogenous monoamine neurotransmitters, such as dopamine and phenylethylamine [2], as well as exogenous amines derived from food and drugs [3]. MAO-B is highly expressed in reactive astrocytes, making it a promising imaging biomarke*r* for neuroinflammation and neurodegenerative diseases [4–6].

Several positron emission tomography (PET) radiotracers have been developed to selectively target MAO-B, ranging from irreversible tracers with short physical half-lives to reversible tracers with long physical half-lives [7–12]. [¹¹C]L-deprenyl and [¹¹C]L-deprenyl-D₂ ([¹¹C]DED) are irreversible tracers, with [¹¹C]DED showing reduced trapping and slower irreversible binding [7]. [¹¹C]SL25.1188 is a reversible MAO-B PET tracer [8] but is also labeled with carbon-11, like the L-deprenyl derivatives, limiting their clinical utility to facilities with onsite cyclotrons. [¹⁸F]F-DED is a fluorine-18-labeled PET tracer, offering logistical advantages over carbon-11-labeled compounds; however, its irreversible binding to MAO-B may limit its applicability in dynamic studies [9]. [¹⁸F]THK-5351, a fluorine-18-labeled PET tracer developed for tau imaging [10], was found to show significant off-target binding to MAO-B [11]. To address this, [¹⁸F]SMBT-1 was developed as a reversible MAO-B tracer through the lead optimization of [¹⁸F]THK-5351 [12]. In brain imaging, [¹⁸F]SMBT-1 revealed a high binding affinity for MAO-B with no off-target interactions with MAO-A, tau, or amyloid [12]. [¹⁸F]SMBT-1 brain imaging has demonstrated increased uptake in patients with Alzheimer’s disease compared to healthy controls, suggesting the presence of reactive astrogliosis associated with early amyloid-beta accumulation [13, 14]. However, numerous studies have demonstrated that MAO-B is not confined to the central nervous system but is also expressed in various peripheral tissues and organs.

Immunohistochemical, autoradiographic, and PET studies have confirmed MAO-B expression in multiple peripheral organs, including the liver, kidneys, heart, and gastrointestinal tissues [15–19]. MAO-B overexpression in the peripheral tissues has been implicated in various inflammatory, immune-mediated, and neoplastic diseases [20, 21]. MAO-B enzymatic activity generates reactive oxygen species (ROS), pro-inflammatory cytokines, and chemokines, which contribute to cellular damage and amplify inflammatory cascades in conditions such as rheumatoid arthritis and inflammatory bowel disease [20]. Elevated MAO-B expression has also been reported in glioblastoma, colorectal, lung, renal, prostate, and bladder cancers, where ROS overproduction may promote tumor progression and immune evasion [21, 22].

PET imaging with MAO-B–specific tracers could offer a powerful tool for assessing MAO-B–mediated oxidative and inflammatory processes in peripheral tissues. Despite promising preclinical results [12], the whole-body distribution of [¹⁸F]SMBT-1 in humans has not yet been established. This study aimed to observe the biodistribution of [¹⁸F]SMBT-1 and evaluate peripheral MAO-B expression in healthy participants using a dynamic PET imaging protocol.

## Materials and methods

### Subjects

Six healthy volunteers (four males and two females; mean age: 39.3 ± 17.4 years) participated in this study. Each participant received an injection of [^18^F]SMBT-1 (168.0 ± 17.8 MBq; Table 1). All participants were healthy, non-smokers, and free of any medically significant abnormalities at the time of scanning. Subjects were recruited based on the following inclusion criteria: aged 20–70 years, physically able to tolerate prolonged scanning sessions lasting 4–5.5 h, and able to remain still throughout the scan. The exclusion criteria were as follows: pregnancy, breastfeeding, or within 28 days postpartum; history of severe allergic reactions; current or history of smoking; contraindications to magnetic resonance imaging (MRI); organ removal or major anatomical abnormalities; severe cardiovascular or systemic disease; liver or kidney dysfunction based on laboratory tests; cancer diagnosis or treatment within the past year; and any condition deemed unsuitable by the investigator. All participants provided written informed consent. The study was approved by the Ethics Committee of Tohoku University Hospital (Database Registration Number: UMIN000041176) and conducted in accordance with the Declaration of Helsinki.

**Table 1:** Participants demographic information

Participants	Age (y)	Gender	Weight (kg)	Height (m)	BMI (kg/m^2^)	Injected Dose (MBq)	Molar Activity (GBq/µmol)
1	22	M	56.0	1.7	20.2	159.0	239.9
2	24	M	61.0	1.7	20.2	189.0	331.7
3	21	M	63.2	1.8	19.8	187.4	291.5
4	52	M	53.8	1.6	20.1	153.9	407.9
5	63	F	59.1	1.5	25.6	177.3	151.1
6	54	F	48.3	1.5	21.9	141.1	80.0
Mean ± SD	**39.3 ± 17.4**		56.9 ± 4.9	1.6 ± 0.1	21.3 ± 2.0	**168.0 ± 17.8**	**250.4 ± 109.6**

F, female; M, male; SD, standard deviation

### Radiotracer synthesis

[¹⁸F]SMBT-1 (Sendai monoamine oxidase B tracer 1; (S)-(2-methylpyrid-5-yl)-6-[(3-¹⁸F-fluoro-2-hydroxy)propoxy]quinoline) was synthesized in-house according to a previously established procedure using the [¹⁸O(p, n)¹⁸F] nuclear reaction [12]. Fluorine-18 was produced by proton bombardment of [¹⁸O]water using a cyclotron, and the resulting [¹⁸F]fluoride was applied in a nucleophilic substitution reaction, in which the radioactive fluoride ion replaced a leaving group on the precursor molecule to yield the labeled intermediate. The reaction mixture subsequently underwent deprotection under acidic conditions to obtain the final active compound. The product was purified by semipreparative high-performance liquid chromatography (HPLC), affording [¹⁸F]SMBT-1 with a radiochemical purity exceeding 95%.

### Metabolite analysis

Blood metabolite data shown in this paper were averaged from individual subject data (*n* = 3) demonstrated in our previous report [23], since blood metabolite analysis was not performed in the present study (Supplementary Table 3, Fig. 7).

### Imaging protocol

**Fig. 1:**
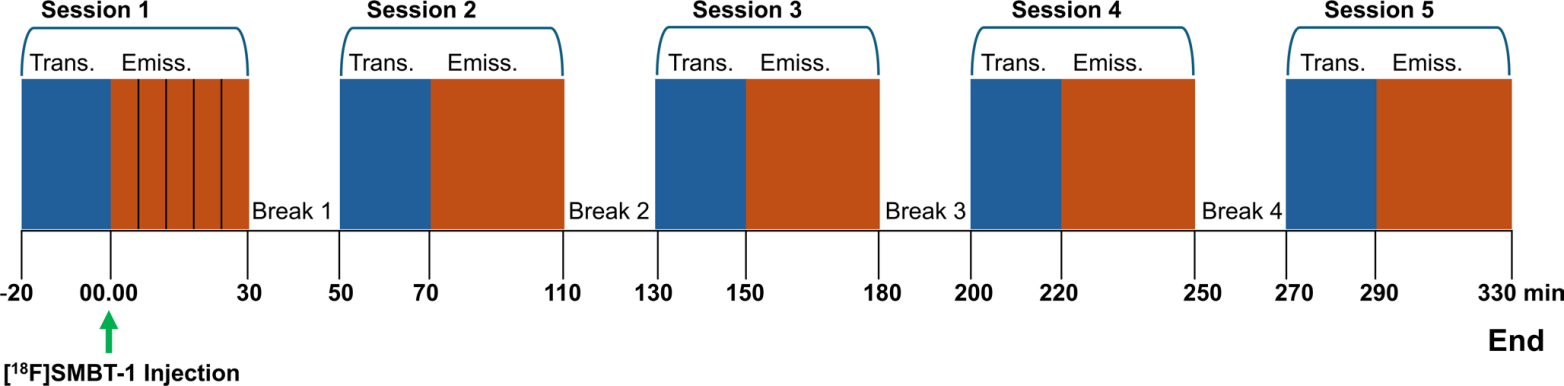
Study design illustrating the time flow of the study. Whole-body dynamic scans were performed in five separate sessions for 330 min following the injection of [^18^F]SMBT-1 (green arrow), with 20-min breaks inserted between each session. Transmission scans (blue) were followed by emission scans (yellow) for each session. Abbreviations: Trans, transmission; Emiss, emission

A series of nine dynamic whole-body PET scans were acquired using an Eminence SET-3000BX PET scanner (Shimadzu Co. Ltd., Kyoto, Japan). PET emission scans were acquired in five sessions over 330 min (min) post-injection (p.i.), covering the vertex to the mid-thigh. The PET scans were divided into five separate sessions as follows: the first session (Session 1), consisting of five consecutive PET scans, was acquired immediately following the [^18^F]SMBT-1 injection from 0 to 30 min p.i. Sessions 2 to 5 were conducted at 70–110, 150–180, 220–250, and 290–330 min p.i., respectively (Fig. 1). Each session was followed by a 20-min break. A 20-min single photon transmission scan using an external ¹³⁷Cs source was performed before the emission scan for attenuation correction. The participants were instructed to void their bladders during the breaks. PET data were corrected for random and scatter coincidences, as well as dead-time losses. The data were reconstructed using the 3D DRAMA algorithm, as recommended by the manufacturer [24]. Images were reconstructed with one iteration and one subset using a field of view (FOV) of 600 mm and a matrix size of 128 × 128 × 337, resulting in voxel dimensions of 4.7 × 4.7 × 3.3 mm³. The resulting image was expressed as kBq/mL. The standardized uptake values (SUVs) were then computed using the following formula: SUV = concentration (in kBq/mL) multiplied by the ratio of body weight (in grams) to the injected dose (in kBq).

MRI was performed using a GE Signa HDxt 1.5T scanner (GE Healthcare, Milwaukee, WI, USA) equipped with the HD28 software. A 3D T1-weighted inversion recovery sequence was acquired with the following imaging parameters: repetition time (TR) = 11.12 ms, echo time (TE) = 4.2 ms, inversion time (TI) = 1800 ms, and flip angle = 10°. The reconstruction field of view (FOV) was 480 mm, with an imaging matrix of 512 × 512 × 120, and voxel dimensions of approximately 0.9 × 0.9 × 9.0 mm³.

### Image analysis

**Fig. 2:**
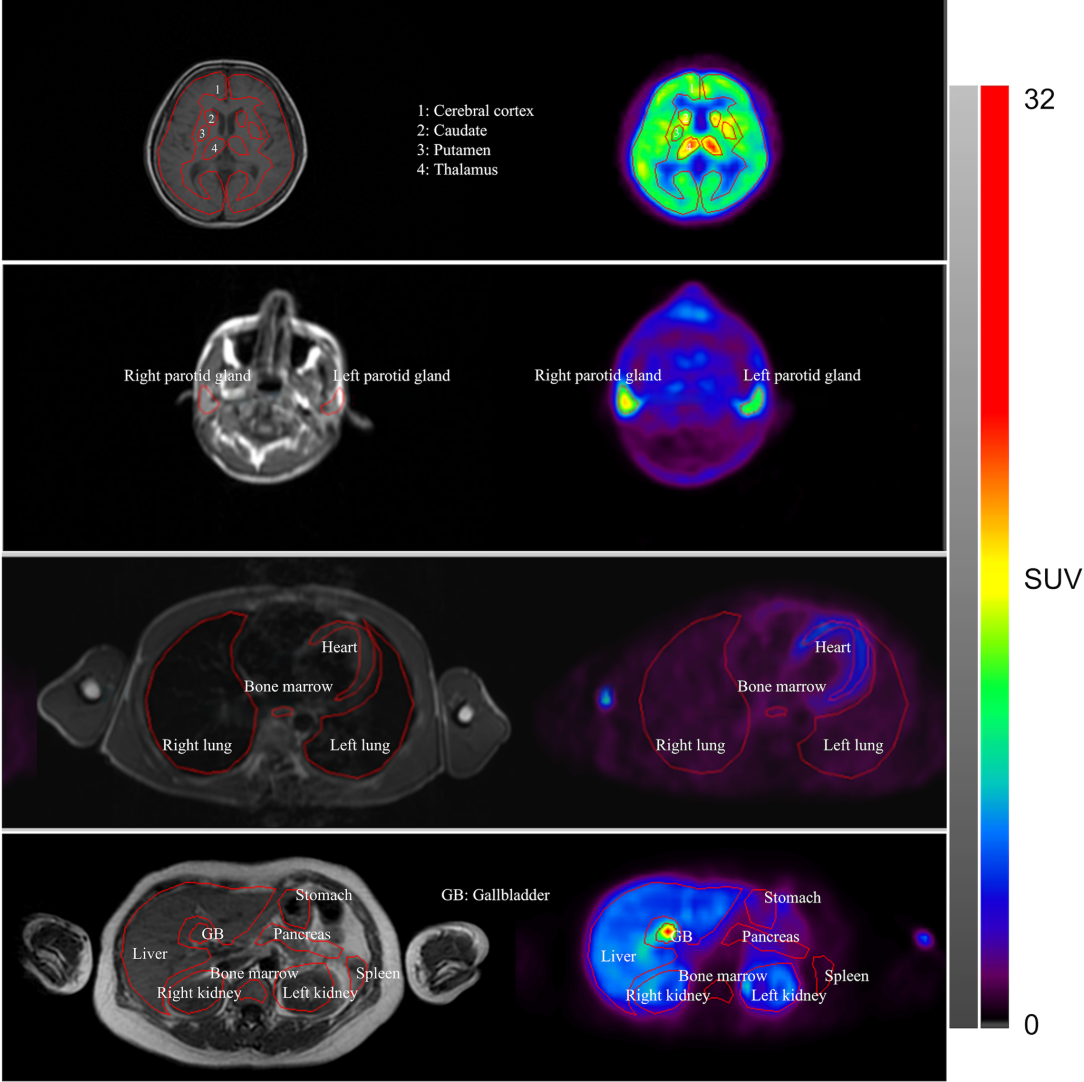
Representative [^18^F]SMBT-1 PET and MR images with examples of region of interest (ROI). In the axial plane, MRI (left) and PET images (right) depict the delineation of interest (ROIs) at the head (brain and parotid glands), chest, and abdominal levels. These ROIs were manually outlined on the PET images, with visual guidance provided by the concurrent display of MRI images on dual monitors, facilitating a side-by-side comparison of both imaging modalities. Abbreviations: MRI, magnetic resonance imaging; ROIs, regions of interest; PET, positron emission tomography

Image analysis was performed using PMOD v4.3 (PMOD Technologies, Zurich, Switzerland) and Dr. View/LINUX R2.5 (AJS Inc., Tokyo, Japan). Following the co-registration of PET images to MRI images in PMOD, regions of interest (ROIs) were manually delineated on PET or MRI using Dr. View/LINUX R2.5 software (Fig. 2). The organs of interest were the brain, submandibular glands, parotid glands, thyroid gland, lungs, breasts, heart, blood pool, kidneys, liver, gallbladder, pancreas, spleen, stomach, small intestine, duodenum, ascending colon, transverse colon, descending colon, rectum, muscles, bone marrow, spinal cord, urinary bladder, vagina, uterus, ovaries, prostate, and testicles. The mean standardized uptake values (SUV_mean_) were extracted for each organ, and time–activity curves (TACs) were generated based on these values.

### Statistical analysis

All statistical analyses were performed using GraphPad Prism 10 (GraphPad Software, Inc. Boston, MA, USA). The Shapiro–Wilk test was used to assess the normality of the SUV distributions. A repeated-measures two-way analysis of variance (ANOVA) was used to determine the changes in the SUVs over time. Tukey’s post hoc correction was applied to adjust for multiple pairwise comparisons and control the risk of Type I errors. Pearson’s correlation analysis was conducted to explore the association in SUV uptake patterns in various organs across subjects.

In addition, a 6 × 6 correlation matrix, based on SUV_mean_ data from 23 organs across nine scans, was generated to illustrate the degree of similarity in whole-body [¹⁸F]SMBT-1 uptake among the six subjects. For this examination, SUV_mean_ values were extracted from 23 predefined organ regions of interest (ROIs) that were identical in both male and female participants across nine post-injection time scans (0–330 min). For bilateral organs (e.g., lungs, kidneys), the mean of the right and left SUV values was used as a single representative value. For each subject, SUV data from all 23 organs and nine time scans (207 total values) were compiled, producing a vector that represents the whole-body distribution pattern of [¹⁸F]SMBT-1 over time. Pearson correlation coefficients were calculated between every pair of subjects (six in total) using these 207-value vectors to quantify the degree of similarity in organ-wise tracer uptake patterns. The resulting 6 × 6 correlation matrix summarizes how closely the spatial and temporal distribution of [¹⁸F]SMBT-1 matched among participants. The matrix is visualized as a color-coded heatmap, where warmer colors indicate higher correlation coefficients.

A mixed-effects model was used to compare [¹⁸F]SMBT-1 uptake across organs in mice and humans, with preclinical data (%ID/g) collected at 2, 10, 30, and 120 min p.i [12]. , and human data (%ID/cc) at 5, 11, 17, 23, 30, and 110 min p.i. These values were analyzed to assess biodistribution differences between species.

## Results

The mean administered activity of [^18^F]SMBT-1 was 168.0 ± 17.8 MBq (range: 141.1–189.0 MBq), and the mean molar activity was 250.4 ± 109.6 GBq/µmol (range: 80.0–407.9 GBq/µmol), as shown in Table 1. No clinically detectable pharmacological effects or significant changes were observed or reported in any of the six subjects. All participants had normal results in their laboratory tests and MRI screenings conducted prior to the PET scans, indicating that they were in good general health at baseline (Table 2).

**Table 2:** Results of renal and liver function tests with normal ranges

Subjects	AST (IU/L)		ALT (IU/L)		Creatinine (mg/dL)	
	Value	Range	Value	Range	Value	Range
ID01	22	10–40	12	5–45	0.6	0.6–1.0
ID02	16	10–40	12	5–45	0.8	0.6–1.0
ID03	15	10–40	15	5–45	0.8	0.6–1.0
ID04	26	10–40	29	5–45	0.9	0.6–1.0
ID05	21	10–40	13	5–45	0.7	0.6–1.0
ID06	17	10–40	11	5–45	0.6	0.6–1.0

ID, identification; AST, aspartate aminotransferase; ALT, alanine aminotransferase; IU/L, international unit per liter; mg/dL, milligram per deciliter

### Biodistribution

**Fig. 3:**
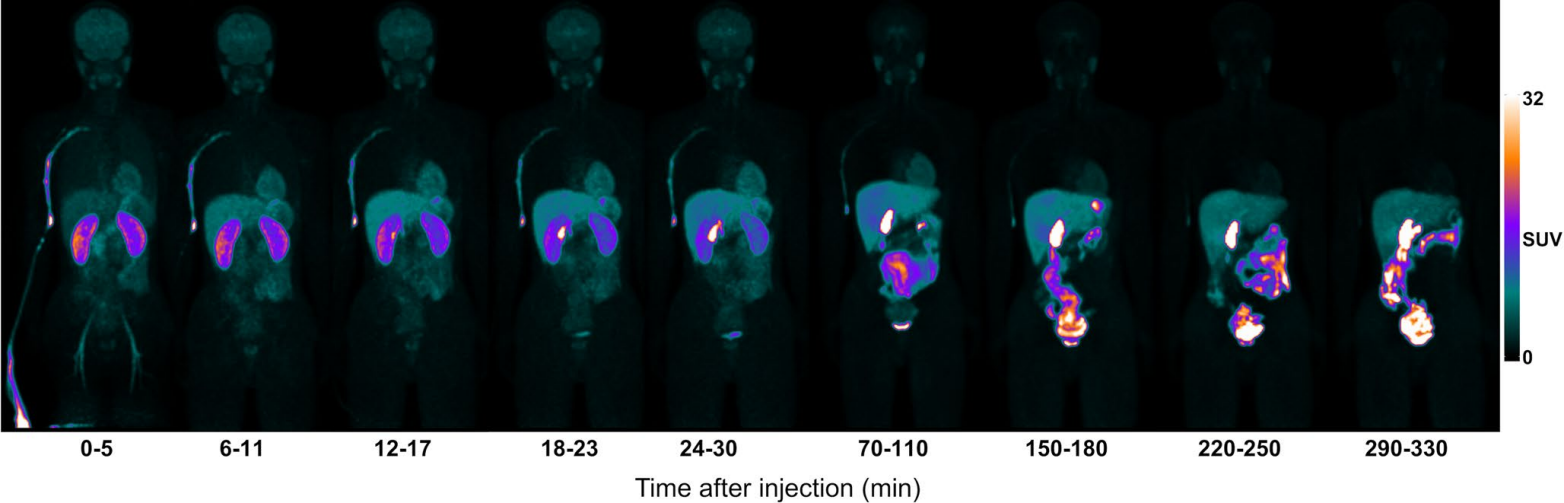
Representative whole-body PET images demonstrating the dynamic biodistribution of [^18^F]SMBT-1 over time. Images are demonstrated in maximum intensity projection (MIP). PET images are demonstrating the dynamic whole-body biodistribution of [^18^F]SMBT-1 to various organs from the early (first 5 min p.i.) to late phases (290 to 330 min p.i.) in a single subject. Early-phase images showed rapid physiological tracer uptake in the kidneys, liver, stomach, heart, spleen, brain, submandibular glands, and parotid glands. Late-phase images showed increased accumulation in the gallbladder, intestine, and urinary bladder. The color scale indicates SUV, ranging from 0 to 32. Abbreviations: MIP, maximum intensity projection; PET, positron emission tomography; SUV, standardized uptake value

**Fig. 4:**
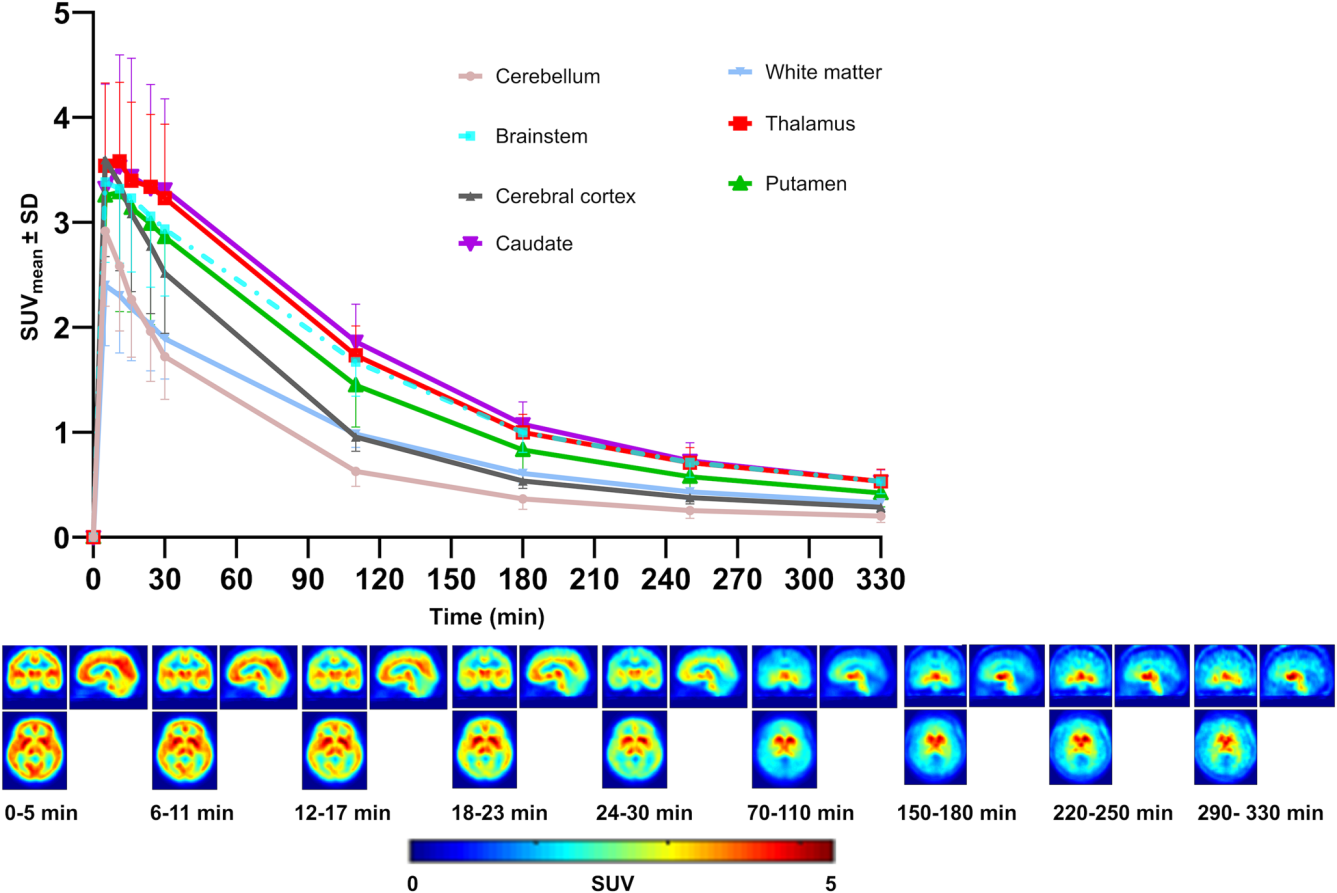
Time-activity curves (TACs) of various brain structures in healthy human subjects. TACs of the mean standardized uptake value (SUV_mean_) ± standard deviation (SD) were derived from six subjects (top panel) and dynamic positron emission tomography (PET) images of the brain averaged from six healthy participants in coronal, sagittal, and transaxial views (bottom panel). The tracer was quickly taken up, reaching its peak between 0 and 5 min p.i., followed by washout from most structures with low MAO-B expression. The cerebellum showed the fastest and highest clearance compared to other regions, possibly indicating very low MAO-B expression. A relatively persistent high uptake with slow clearance was observed in the caudate, thalamus, and midbrain for up to 330 min p.i., indicating high MAO-B expression

Early uptake of [^18^F]SMBT-1 was observed in the kidneys, liver, stomach, heart, spleen, brain, and salivary glands, with subsequent redistribution predominantly to the gallbladder, intestine, and urinary bladder (Fig. 3, Supplementary Table 1). Following [¹⁸F]SMBT-1 injection, strong early brain uptake was observed in the cortical and subcortical gray matter within the first 30 min p.i. The cortical regions’ uptake was cleared starting from approximately 70 min p.i., while medium retention persisted in the basal ganglia, thalamus, and midbrain (Fig. 4).

**Fig. 5:**
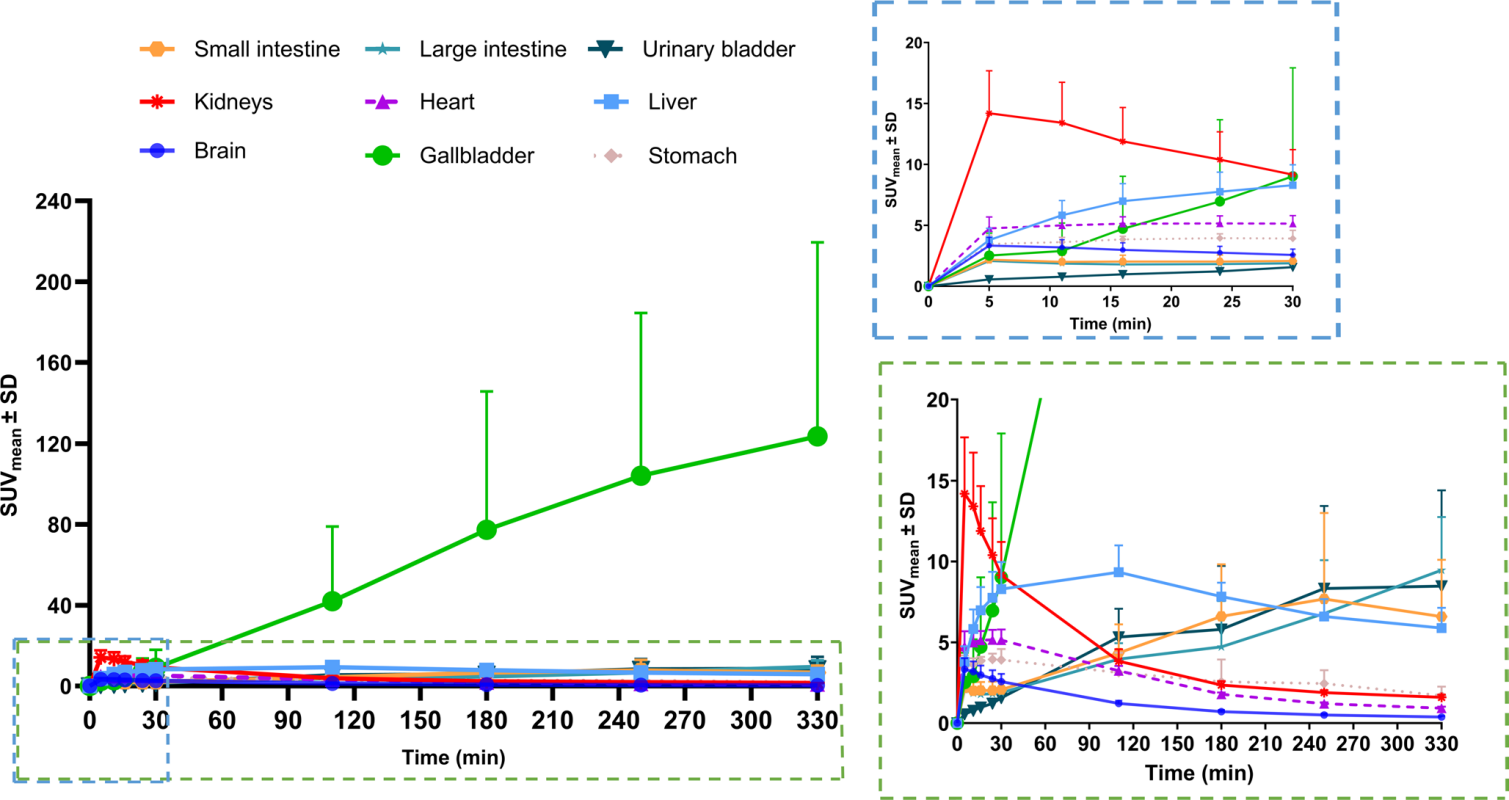
Time-activity curves (TACs) of various organs in healthy human subjects. The TACs of the mean standardized uptake value (SUV_mean_) ± standard deviation (SD) derived from six subjects demonstrated high uptake in the gallbladder. An early uptake was observed in the kidneys, heart, liver, brain, and stomach from 0 to 30 min p.i., as indicated in the right upper, blue-dashed box. A subsequent late uptake is depicted in the right bottom, marked by a green-dashed box, which encompasses the intestine and urinary bladder

The TACs of SUV_mean_ averaged from six subjects also indicated an early peak followed by washout in the brain, kidneys, heart, and stomach (Fig. 5). The brain, kidneys, heart, and stomach exhibited peak activity within 5 min p.i., with the kidneys demonstrating the highest uptake (SUV_mean_ = 14.2 ± 3.5; Fig. 5, Supplementary Table 1). The uptake in these organs plateaued until 30 min p.i., after which a progressive decline was observed. The liver exhibited a gradual uptake, peaking at 100 min (SUV_mean_ = 9.3 ± 1.7), followed by a steady decline *(*Fig. 5, Supplementary Table 1). Gallbladder uptake progressively increased from 30 min p.i., reaching the highest late-phase value (SUV_mean_ = 123.7 ± 53.4 at 310 min) (Fig. 5, Supplementary Table 1). The urinary bladder, small intestine, and large intestine exhibited a late-phase increase in [¹⁸F]SMBT-1 uptake (Fig. 5). Gallbladder uptake was approximately tenfold higher than that of the urinary bladder, indicating predominant hepatobiliary excretion.

**Fig. 6:**
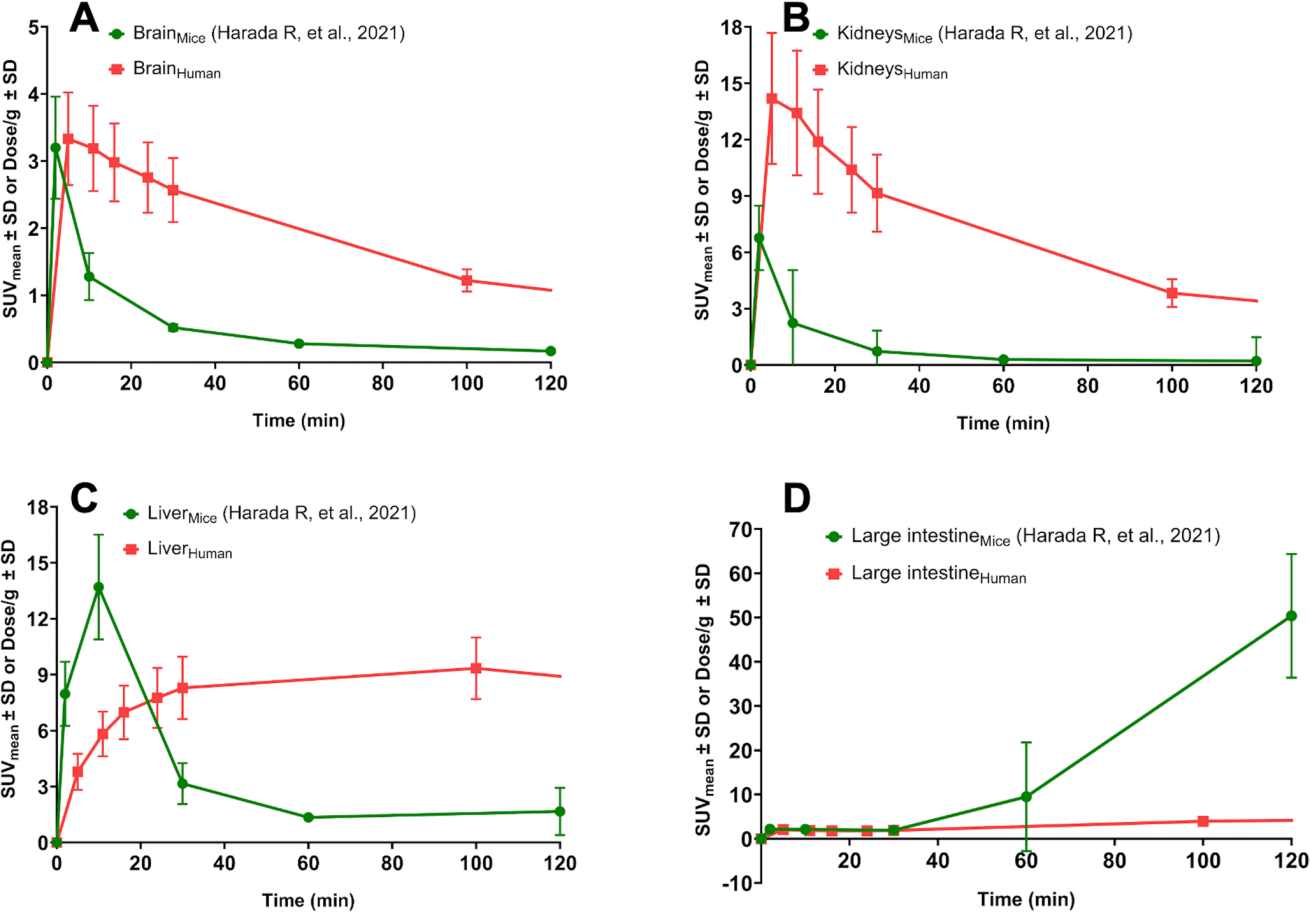
Time-activity curves (TACs) of various organs in mice and healthy human subjects. The time-activity curves (TACs) of the standardized uptake value mean (SUV_mean_) ± standard deviation (SD) in humans, alongside the dose per gram ± standard deviation (SD) in mice, illustrate the comparative uptake patterns of [¹⁸F]SMBT-1. Comparable trends were observed in the brain (**A**) and kidneys (**B**), where both species showed early tracer uptake. In contrast, the liver (**C**) and intestines (**D**) exhibited earlier retention in mice than in humans

Human [¹⁸F]SMBT-1 biodistribution data were compared with previously reported preclinical findings in mice [12] across representative organs, including the brain, kidneys, liver, and large intestine (Fig. 6). Similar early uptake patterns were observed in the brain (Fig. 6A) and kidneys (Fig. 6B) in both species. In contrast, the liver (Fig. 6C) and large intestine (Fig. 6D) exhibited earlier and more pronounced retention in mice, suggesting species-specific differences in the rates of tracer metabolism and excretion. There was no significant difference in uptake pattern between human and preclinical mice data (*p* = 0.39).

Two-way repeated measures ANOVA using SUV_mean_ values from 23 organs across nine time points showed a significant effect of time on tracer distribution (*p* = 0.01), with no significant difference between the six participants (*p* = 0.93) and between sex and age groups (*p* > 0.99). Significant positive correlations in [^18^F]SMBT-1 uptake patterns were observed in most target organs between male and female participants (*r* > 0.84, *p* < 0.004) and between middle-aged and young participants (*r* > 0.85, *p* < 0.004), except in the intestine (Supplementary Table 2). A Pearson correlation matrix constructed based on the SUV_mean_ values obtained from 23 organs across nine sequential time points revealed strong inter-subject correlations in whole-body tracer distribution and clearance patterns (*r* = 0.87–0.99, *p* < 0.001; Supplementary Fig. 1).

**Fig. 7:**
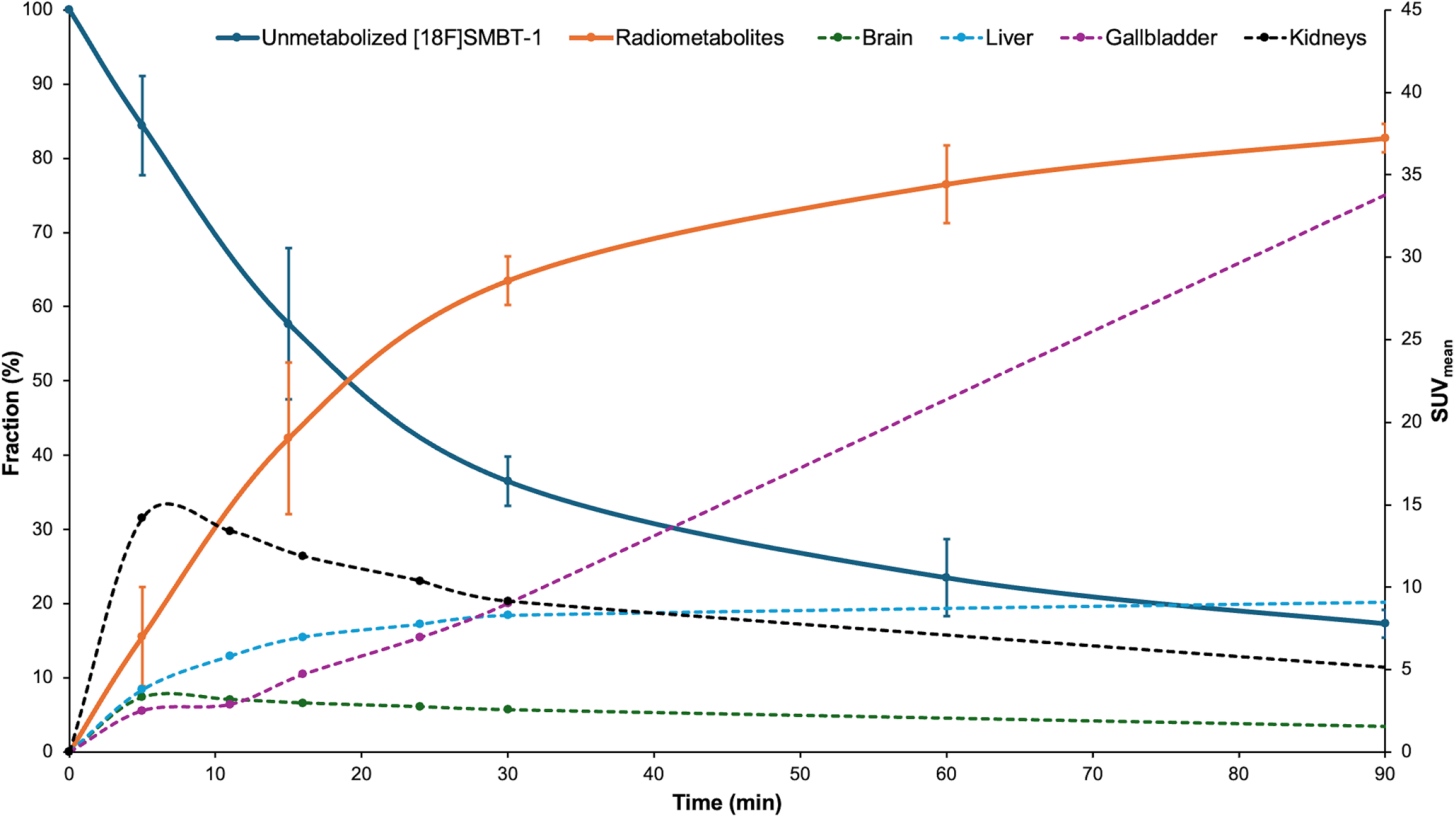
Time course of unmetabolized and metabolized [^18^F]SMBT-1 fraction. The mean fraction data of unmetabolized and metabolized [^18^F]SMBT-1 were averaged from three healthy subjects in the previous study [33], as well as average SUV_mean_ values for selected regions of interest, including the brain, kidneys, gallbladder, and liver in the six healthy subjects in this study. The left Y-axis indicates the plasma fractions of parent [^18^F]SMBT-1 (solid lines) and its radiometabolites, whereas the right Y-axis shows the SUV_mean_ of each representative organ (dotted lines). The X-axis represents time post-injection of [^18^F]SMBT-1. Error bars indicate standard deviation

As for the metabolite data, the mean parent fraction of unmetabolized [¹⁸F]SMBT-1 at 5, 15, 30, 60, and 90 min p.i. was 84.4%, 57.7%, 36.5%, 23.5%, and 17.3%, respectively [23], as shown in Supplementary Tables 3 and Fig. 7.

## Discussion

Early uptake of [¹⁸F]SMBT-1 in multiple organs, including the kidneys, heart, liver, pancreas, stomach, duodenum, and spleen, reflects the known patterns of MAO-B expression, consistent with previous immunohistochemical, autoradiographic, and PET tracer studies [15–19]. However, the rapid peak and subsequent decline may also reflect regional blood flow effects, not solely MAO-B binding. A similar high early uptake has been reported shortly after injection with other tracers, such as FDG, reflecting blood flow–dependent delivery and providing an indirect estimate of perfusion, similar to that of [¹⁵O]-labeled water [25]. Both [¹⁸F]SMBT-1 and the irreversible tracer [¹¹C]DED displayed a comparable pattern of a rapid peak and a decline, followed by a sustained plateau in [¹¹C]DED [18], while showing a slow washout with [¹⁸F]SMBT-1. The plateau noted in [¹¹C]DED studies may indicate irreversibly trapped selective binding to MAO-B as an index correlated with MAO-B density. Similarly, the slow washout observed with [¹⁸F]SMBT-1 may indicate MAO-B density in these organs. The advantage of [¹⁸F]SMBT-1 over [¹¹C]DED is that it allows for long-duration dynamic scanning.

MAO-B catalyzes the degradation of dopamine, histamine, tyramine, and other monoamines in organs such as the kidneys [26], heart [27], and gastrointestinal tract [28]. By producing hydrogen peroxide as a metabolic byproduct, MAO-B enzymatic activity initiates the formation of reactive oxygen species (ROS), which in turn drives oxidative stress associated with the development of heart and renal failure, as well as inflammatory bowel disease [26–28]. Furthermore, MAO-B contributes to tumor progression by upregulating pro-inflammatory mediators and promoting oxidative stress, thereby supporting malignant transformation and growth [21, 22]. MAO-B-targeted PET tracers have been used to assess peripheral MAO-B expression [18] and investigate the biological impact of smoking on MAO-B [19]. In a separate pathological context, autoradiography using [³H]lazabemide, a selective radiolabeled MAO-B tracer, revealed high peripheral MAO-B expression in the heart, lung, liver, kidney, spleen, and duodenum using postmortem tissue samples from individuals who died of chronic renal insufficiency, pericarditis, Alzheimer’s disease, pneumonia, or melanoma [15]. [¹⁸F]SMBT-1, with its favorable kinetics and MAO-B selectivity, could also enable detection of peripheral inflammatory activities associated with high MAO-B expression. The increased MAO-B activity could be also observed in brain tumors such as glioblastoma (GBM) using [¹⁸F]THK5351 [29–33], suggesting further potential applicability of [¹⁸F]SMBT-1 for detecting GBM and other MAO-B–related glial changes in tumor tissues.

In the brain, [¹⁸F]SMBT-1 demonstrated rapid uptake and region-specific retention, consistent with previous findings [12, 13]. This reflects the efficient blood-brain barrier permeability of [¹⁸F]SMBT-1 [12]. Increased elimination was observed in regions with low MAO-B activity, such as the cerebral cortex and cerebellum, and persistent late retention in the basal ganglia, thalamus, and midbrain likely represents physiological MAO-B expression in non-reactive astrocytes. No significant inter-subject variation in brain uptake was observed in our healthy cohort, despite prior reports of age-related increases in retention between 60 and 80 min [13]. The specificity of the tracer was confirmed by blocking studies with selective MAO-B inhibitors, in which over 85% of the brain signal was eliminated after selegiline administration [13]. These findings support the use of [¹⁸F]SMBT-1 as a reliable and selective tracer for imaging astrogliosis in neurodegenerative and inflammatory brain disorders.

Minimal [¹⁸F]SMBT-1 uptake in the thyroid gland aligns with previous studies that showed a lack of MAO-B expression in thyroid follicular and parafollicular cells [16]. Although [¹⁸F]SMBT-1 uptake is high in the salivary glands, there is no definitive evidence of MAO-B expression in these tissues. This uptake may reflect high perfusion despite prolonged retention, suggesting mechanisms beyond perfusion.

[¹⁸F]SMBT-1 is metabolized in the liver via sulfate conjugation [34], catalyzed by hepatic sulfotransferases, primarily sulfotransferase family 1A member 1 (SULT1A1), which is abundantly expressed in the human liver [35]. The major radiometabolite, O-sulfated [¹⁸F]SMBT-1 [12, 34], is excreted into bile, passes through the common bile duct into the duodenum, and progresses through the intestinal tract. The gallbladder consistently showed the highest SUV values during the late phase, reflecting its role as a temporary storage site for radiometabolites prior to their release into the intestine. The administration of a small amount of dietary fat supplement after injection of [^18^F]SMBT-1 could be explored as a strategy to promote bile flow and reduce tracer retention in the gallbladder [36]. In a previous study, a 71.3% reduction in gallbladder dose was observed after taking a fatty meal supplement [36]. A similar biodistribution pattern was observed in a preclinical mice study, with delayed intestinal accumulation and a more rapid decline in liver retention compared to humans. The more rapid liver washout in mice may reflect an accelerated metabolism driven by increased liver enzyme activity [37].

Because this protocol required multiple whole-body acquisitions in healthy volunteers, it was crucial to minimize the additional radiation exposure from transmission scans. For this reason, we employed a ¹³⁷Cs-based transmission system, which delivers substantially lower radiation doses than CT-based transmission imaging [38, 39]. Previous studies have demonstrated that radionuclide-based transmission sources, such as ⁶⁸Ge, delivered negligible effective doses (< 0.3 mSv per session) [38], and that point-source ¹³⁷Cs transmission measurements resulted in ~ 0.011 mSv [39]. Meanwhile, CT-based transmission scans have been reported to yield effective doses of ~ 8.8 mSv (high-speed mode) per session [38]. The whole-body biodistribution data obtained in this study will be further utilized for radiation dosimetry analysis, which will be published in a separate report. Additionally, these data may serve as a valuable baseline for future investigations involving peripheral [¹⁸F]SMBT-1 imaging.

The primary limitation of this study was its small sample size (*n* = 6), which may have reduced the statistical power and limited the generalizability of the findings. Furthermore, although [¹⁸F]SMBT-1 demonstrated clear uptake in MAO-B–rich regions, the possibility that radiometabolites contributed to the observed PET signal cannot be completely excluded. Our precious clinical data indicated that metabolites accounted for approximately 64% of total radioactivity in healthy humans at 30 min p.i [23]. , and a previous preclinical plasma analysis demonstrated that approximately 80% of [¹⁸F]SMBT-1 was metabolized at 30 min p.i [12]. Brain would be the only organ without binding of metabolized [¹⁸F]SMBT-1 because of the blood-brain barrier (BBB) blocking the penetration of highly-polarized metabolites [12, 34], whereas uptake in the gallbladder appears to represent almost entirely metabolized [¹⁸F]SMBT-1. In other peripheral organs, early-phase uptake is largely driven by MAO-B binding of unmetabolized [¹⁸F]SMBT-1, though late-phase uptake likely reflects a mixture of unmetabolized and metabolized tracer. Accordingly, the relative contributions of unmetabolized to metabolized [¹⁸F]SMBT-1 are expected to vary across organs depending on their perfusion, metabolic activity, and excretory function.

## Conclusion

This study provided the first human whole-body distribution map of [¹⁸F]SMBT-1, demonstrating its favorable pharmacokinetic properties for brain imaging. The observed peripheral uptake patterns suggest the potential for future applications in imaging peripheral MAO-B expression in systemic inflammatory and immune-mediated diseases. These data may also serve as a baseline for comparison in future studies. Gallbladder retention findings indicated the need for practical imaging strategies to reduce tracer accumulation, such as administering a dietary fat supplement after [¹⁸F]SMBT-1 injection.

## Electronic Supplementary Material

Below is the link to the electronic supplementary material.

Supplementary Material 1
