# Supplementary material for: Whole-body biodistribution of [18F]SMBT-1: a novel PET tracer for monoamine oxidase B imaging in healthy humans
Source: Ann Nucl Med. 2026 Jan 7;40(4):405–15. doi: 10.1007/s12149-025-02144-2 (PMC13032976; doi:10.1007/s12149-025-02144-2)

Supplementary materials to:

**Whole-Body Biodistribution of [^18^F]SMBT-1: A Novel PET Tracer for Monoamine Oxidase B Imaging in Healthy Humans**

**Authors Name:**

Berihu Mesfin^1^, Yui Ishioka^1^, Yoshiki Ichinose^1^, Akihito Inamura^2^, Yingying Wu^1^, Shoichi Watanuki^1,4^, Kotaro Hiraoka^1,4^, Yoshihito Funaki^3^, Asuka Kikuchi^1,4^, Kazuko Takeda^1,3^, Masayasu Miyake^2^, Ryuichi Harada^3,5,6^, Shozo Furumoto^3,4^, Nobuyuki Okamura^5^, Kazuhiko Yanai^1,6^, Hiroshi Watabe^2,4^, Manabu Tashiro^1,4^

**Affiliations:**

^1^Nuclear Medicine Laboratory, Research Center for Accelerator and Radioisotope Science, Tohoku University, Sendai, Japan

^2^Radiation Protection and Safety Control Laboratory, Research Center for Accelerator and Radioisotope Science, Tohoku University, Sendai, Japan

^3^Radiopharmaceutical Laboratory, Research Center for Accelerator and Radioisotope Science, Tohoku University, Sendai, Japan.

^4^Department of Diagnostic Radiology, Tohoku University Hospital, Tohoku University, Sendai, Japan

^5^Division of Pharmacology, Faculty of Medicine, Tohoku Medical and Pharmaceutical University, Sendai, Japan

^6^Department of Pharmacology, Graduate School of Medicine, Tohoku University, Sendai, Japan

**Correspondence**: Manabu Tashiro, MD, PhD (manabu.tashiro.a2@tohoku.ac.jp)

| **Supplementary Table 1.** Time-Dependent Changes in [^18^F]SMBT-1 Uptake Across Different Organs | | | | | | | | | |
| --- | --- | --- | --- | --- | --- | --- | --- | --- | --- |
| **Target Organs** | **0-5 min** | **6-11 min** | **12-17 min** | **18-23-min** | **24-30 min** | **70-110 min** | **150-180 min** | **220-250 min** | **290-330 min** |
| Brain | **3.3 ± 0.7** | **3.2 ± 0.6** | **3.0 ± 0.6** | **2.8 ± 0.5** | **2.6 ± 0.5** | 1.2 ± 0.2 | 0.7 ± 0.1 | 0.5 ± 0.1 | 0.4 ± 0.1 |
| SMG | 3.4 ± 0.9 | 3.6 ± 0.8 | 3.7 ± 0.9 | 3.8 ± 0.8 | 3.8 ± 0.8 | 2.9 ± 0.6 | 1.7 ± 0.4 | 1.3 ± 0.3 | 0.8 ± 0.3 |
| Parotid glands | 2.4 ± 0.6 | 2.6 ± 0.6 | 2.8 ± 0.7 | 2.9 ± 0.7 | 2.9 ± 0.7 | 2.7 ± 0.6 | 1.8 ± 0.5 | 1.5 ± 0.3 | 1.1 ± 0.3 |
| Lungs | 2.0 ± 0.8 | 1.3 ± 0.6 | 1.0 ± 0.4 | 0.9 ± 0.4 | 0.8 ± 0.4 | 0.5 ± 0.2 | 0.4 ± 0.2 | 0.3 ± 0.1 | 0.2 ± 0.1 |
| Heart | 4.9 ± 1.0 | 5.0 ± 0.7 | 5.1 ± 0.6 | 5.2 ± 0.6 | 5.1 ± 0.7 | 3.2 ± 0.3 | 1.8 ± 0.1 | 1.2 ± 0.1 | 0.9 ± 0.1 |
| Blood pool | 2.2 ± 1.0 | 2.3 ± 1.1 | 2.2 ± 1.1 | 2.2 ± 1.1 | 2.2 ± 1.0 | 1.4 ± 0.5 | 1.0 ± 0.3 | 0.7 ± 0.2 | 0.6 ± 0.2 |
| Esophagus | 1.5 ± 0.5 | 1.4 ± 0.5 | 1.3 ± 0.4 | 1.3 ± 0.5 | 1.2 ± 0.5 | 0.8 ± 0.3 | 0.8 ± 0.7 | 0.6 ± 0.3 | 0.5 ± 0.2 |
| Stomach | 3.4 ± 0.7 | 3.6 ± 0.4 | 3.8 ± 0.2 | 3.9 ± 0.4 | 3.9 ± 0.7 | 3.1 ± 1.2 | 2.6 ± 1.4 | 2.5 ± 0.8 | 1.7 ± 0.6 |
| Spleen | 3.5 ± 0.8 | 1.7 ±0 .3 | 1.4 ± 0.2 | 1.2 ± 0.2 | 1.2 ± 0.2 | 0.8 ± 0.1 | 0.7 ± 0.1 | 0.5 ± 0.1 | 0.6 ± 0.2 |
| Liver | 3.8 ± 1.0 | 5.8 ± 1.2 | 7.0 ± 1.4 | 7.8 ± 1.6 | 8.3 ± 1.7 | **9.3 ± 1.7** | 7.8 ± 0.9 | 6.6 ± 1.1 | 5.9 ±1.3 |
| Gallbladder | 2.5 ± 0.4 | 2.9 ± 0.4 | 4.7 ± 1.3 | 7.0 ± 2.4 | 9.0 ± 4.2 | 42.1 ± 24.5 | 77.4 ± 50.2 | 104.1 ± 49.0 | **123.7 ± 53.4** |
| Pancreas | 5.5 ± 1.4 | 4.6 ± 1.3 | 4.0 ± 1.0 | 3.8 ± 1.0 | 3.7 ± 1.1 | 3.1 ± 1.6 | 1.9 ± 1.0 | 1.8 ± 0.9 | 1.7 ± 0.6 |
| Duodenum | 4.1 ± 1.5 | 3.1 ± 1.4 | 3.1 ± 1.4 | 3.1 ± 1.3 | 3.4 ± 1.9 | 5.7 ± 4.1 | 5.7 ± 3.1 | 3.5 ± 1.7 | 4.9 ± 5.7 |
| Small Intestine | 2.2 ± 0.6 | 2.0 ± 0.5 | 2.0 ± 0.5 | 2.0 ± 0.6 | 2.1 ± 0.5 | 4.4 ± 1.8 | 6.6 ± 3.2 | 7.7 ± 5.3 | 6.6 ± 3.5 |
| Ascending colon | 1.4 ± 0.4 | 1.3 ± 0.3 | 1.2 ± 0.3 | 1.2 ± 0.3 | 1.2 ± 0.3 | 1.4 ± 0.6 | 3.4 ± 2.7 | 3.6 ± 2.9 | 6.7 ± 3.1 |
| Transverse colon | 2.1 ± 0.4 | 1.9 ± 0.2 | 1.8 ± 0.3 | 1.8 ± 0.3 | 1.9 ± 0.4 | 4.0 ± 1.3 | 4.7 ± 3.9 | 5.5 ± 2.6 | 5.4 ± 1.3 |
| Descending colon | 1.8 ± 0.5 | 1.8 ± 0.5 | 1.8 ± 0.5 | 1.8 ± 0.5 | 1.9 ± 0.4 | 3.1 ± 1.3 | 4.0 ± 1.8 | 6.8 ± 3.4 | 9.5 ± 4.2 |
| Rectum | 0.7 ± 0.4 | 1.1 ± 0.2 | 1.1 ± 0.2 | 1.1 ± 0.2 | 1.1 ± 0.2 | 1.2 ± 0.8 | 2.1 ± 3.0 | 2.6 ± 4.3 | 2.7 ± 4.6 |
| Kidneys | **14.2 ± 3.5** | 13.4 ± 3.3 | 11.9 ± 2.8 | 10.4 ± 2.3 | 9.2 ± 2.1 | 3.8 ± 0.7 | 2.4 ± 0.3 | 1.9 ± 0.2 | 1.6 ± 0.2 |
| Urinary bladder | 0.6 ± 0.2 | 0.8 ± 0.1 | 1.0 ± 0.2 | 1.2 ± 0.3 | 1.6 ± 0.3 | 5.3 ± 1.8 | 5.8 ± 3.9 | 8.3 ± 5.1 | 8.5 ± 5.9 |
| Muscles | 0.4 ± 0.1 | 0.6 ± 0.2 | 0.7 ± 0.2 | 0.7 ± 0.2 | 0.7 ± 0.2 | 0.8 ± 0.1 | 0.7 ± 0.1 | 0.7 ± 0.1 | 06 ± 0.1 |
| Bone marrow | 0.8 ± 0.2 | 0.9 ± 0.2 | 0.8 ± 0.2 | 0.8 ± 0.2 | 0.8 ± 0.2 | 0.5 ± 0.1 | 0.5 ± 0.1 | 0.5 ± 0.1 | 0.5 ± 0.1 |
| Spinal cord | 0.9 ± 0.3 | 0.9 ± 0.3 | 0.9 ± 0.2 | 0.8 ± 0.2 | 0.8 ± 0.2 | 0.4 ± 0.1 | 0.4 ± 0.1 | 0.4 ± 0.1 | 0.4 ± 0.1 |
| Thyroid | 1.2 ± 0.5 | 1.0 ± 0.4 | 0.9 ± 0.4 | 0.8 ± 0.3 | 0.8 ± 0.3 | 0.6 ± 0.2 | 0.5 ± 0.2 | 0.4 ± 0.1 | 0.3 ± 0.1 |
| Prostate | 0.6 ± 0.3 | 1.4 ± 0.5 | 1.3 ± 0.4 | 1.3 ± 0.3 | 1.2 ± 0.3 | 1.3 ± 0.2 | 1.1 ± 0.2 | 1.0 ± 0.2 | 0.9 ± 0.1 |
| Testicles | 0.1 ± 0.0 | 0.4 ± 0.0 | 0.4 ± 0.0 | 0.4 ± 0.1 | 0.4 ± 0.1 | 0.5 ± 0.1 | 0.4 ± 0.1 | 0.4 ± 0.1 | 0.4 ± 0.1 |
| Breasts | 0.5 ± 0.0 | 0.5 ± 0.1 | 0.5 ± 0.1 | 0.5 ± 0.1 | 0.5 ± 0.1 | 0.3 ± 0.1 | 0.4 ± 0.1 | 0.4 ± 0.1 | 0.4 ± 0.1 |
| Ovaries | 1.3 ± 0.0 | 1.3 ± 0.0 | 1.1 ± 0.0 | 1.2 ± 0.0 | 1.2 ± 0.1 | 1.2 ±0 .3 | 1.0 ± 0.2 | 1.1 ± 0.2 | 0.9 ± 0.1 |
| Uterus | 2.0 ± 0.60 | 2.2 ± 0.7 | 2.2 ± 0.6 | 2.2 ± 0.5 | 2.3 ± 0.6 | 2.3 ± 0.6 | 2.3 ± 0.7 | 1.6 ± 0.2 | 1.4 ± 0.3 |
| Vagina | 0.1 ± 0.2 | 1.1 ± 0.2 | 1.4 ± 0.0 | 1.4 ± 0.1 | 1.6 ± 0.2 | 1.7 ± 0.1 | 1.8 ± 0.2 | 1.8 ± 0.2 | 1.6 ± 0.3 |

^Two-way ANOVA (Organ × Time): p < 0.0001.^

^Values represent averaged SUVmean ± SD from six subjects.^

**^Abbreviations:^** ^SUVmean, mean standardized uptake value; SMG, submandibular gland; SD, standard deviation^

**Supplementary Table 2.** Pearson Correlation Analysis of [¹⁸F]SMBT-1 Uptake Trends Stratified by Sex and Age.

| **Target Organs** | **Sex Group (Male n = 4, Female n = 2)** | | **Age Group (Young n = 3, Middle-Aged n = 3)** | |
| --- | --- | --- | --- | --- |
|  | Pearson's correlation (r) | Significance p-value | Pearson's correlation (r) | Significance p-value |
| Brain | 0.99 | 0.0001 | 0.99 | 0.0001*** |
| Submandibular glands | 0.98 | 0.0001 | 0.98 | 0.0001*** |
| Parotid glands | 0.99 | 0.0001 | 0.99 | 0.0001*** |
| Lungs | 0.99 | 0.0001 | 0.99 | 0.0001 |
| Heart | 0.97 | 0.0001 | 0.99 | 0.0001 |
| Blood pool | 0.99 | 0.0001 | 0.99 | 0.0001 |
| Esophagus | 0.92 | 0.0003 | 0.97 | 0.0001 |
| Stomach | 0.84 | 0.0044 | 0.85 | 0.0042 |
| Spleen | 0.99 | 0.0001 | 0.99 | 0.0001 |
| Liver | 0.88 | 0.0019 | 0.97 | 0.0001 |
| Gallbladder | 0.98 | 0.0001 | 0.99 | 0.0001 |
| Pancreas | 0.98 | 0.0001 | 0.98 | 0.0001 |
| Duodenum | 0.12 | 0.7529* | 0.48 | 0.1988* |
| Small intestine | 0.94 | 0.0002 | 0.95 | 0.0001 |
| Ascending colon | 0.78 | 0.0131 | 0.84 | 0.0047 |
| Transverse colon | 0.59 | 0.0977* | 0.90 | 0.0008 |
| Descending colon | 0.90 | 0.0007 | 0.95 | 0.0001 |
| Rectum | -0.63 | 0.0663* | -0.53 | 0.139* |
| Kidneys | 0.99 | 0.0001 | 0.99 | 0.0001 |
| Urinary bladder | 0.95 | 0.0001 | 0.96 | 0.0001 |
| Muscles | 0.93 | 0.0003 | 0.95 | 0.0001 |
| Bone marrow | 0.98 | 0.0001 | 0.99 | 0.0001 |
| Thyroid | 0.92 | 0.0003 | 0.95 | 0.0001 |
| **Overall** | **0.98** | **0.0001** | **0.99** | **0.0001** |
| * p < 0.05, ** p < 0.001, ***p < 0.0001 | | | | |

**Supplementary Table 3.** Time-Dependent Changes in the Fraction of Unmetabolized and Metabolized [^18^F]SMBT-1 in Healthy Human Subjects (Hiraoka K, et al., 2025)

| Time | Unmetabolized [^18^F]SMBT-1 | | Metabolites | |
| --- | --- | --- | --- | --- |
|  | Percent (%) | S.D. | Percent (%) | S.D. |
| 5 | 84.4 | 6.7 | 15.6 | 6.7 |
| 15 | 57.7 | 10.2 | 42.3 | 10.2 |
| 30 | 36.5 | 3.3 | 63.5 | 3.3 |
| 60 | 23.5 | 5.2 | 76.5 | 5.2 |
| 90 | 17.3 | 1.9 | 82.7 | 1.9 |

^The data were averaged from individual subject data (n = 3). S.D., standard deviation^

**Supplementary Fig. 1**. Heatmap of the Pearson correlation matrix illustrating inter-subject similarity in whole-body [¹⁸F]SMBT-1 uptake patterns across six subjects (ID01–ID06). All correlation coefficients exceeded 0.87, showing a strong association between the distribution patterns across the subjects. All correlations were statistically significant (p < 0.001).

Standardized uptake values (SUVs) were extracted from 23 predefined organ regions of interest (ROIs) that were identical in both male and female participants across nine post-injection time scans (0–90 min). For each subject, SUV_mean_ data from all 23 organs and nine time scans (207 total values) were compiled, producing a vector that represents the whole-body distribution pattern of [¹⁸F]SMBT-1 over time. Pearson correlation coefficients were calculated between every pair of subjects (six in total) using these 207-value vectors to quantify the degree of similarity in organ-wise tracer uptake patterns. The resulting 6 × 6 correlation matrix summarizes how closely the spatial and temporal distribution of [¹⁸F]SMBT-1 matched among participants.


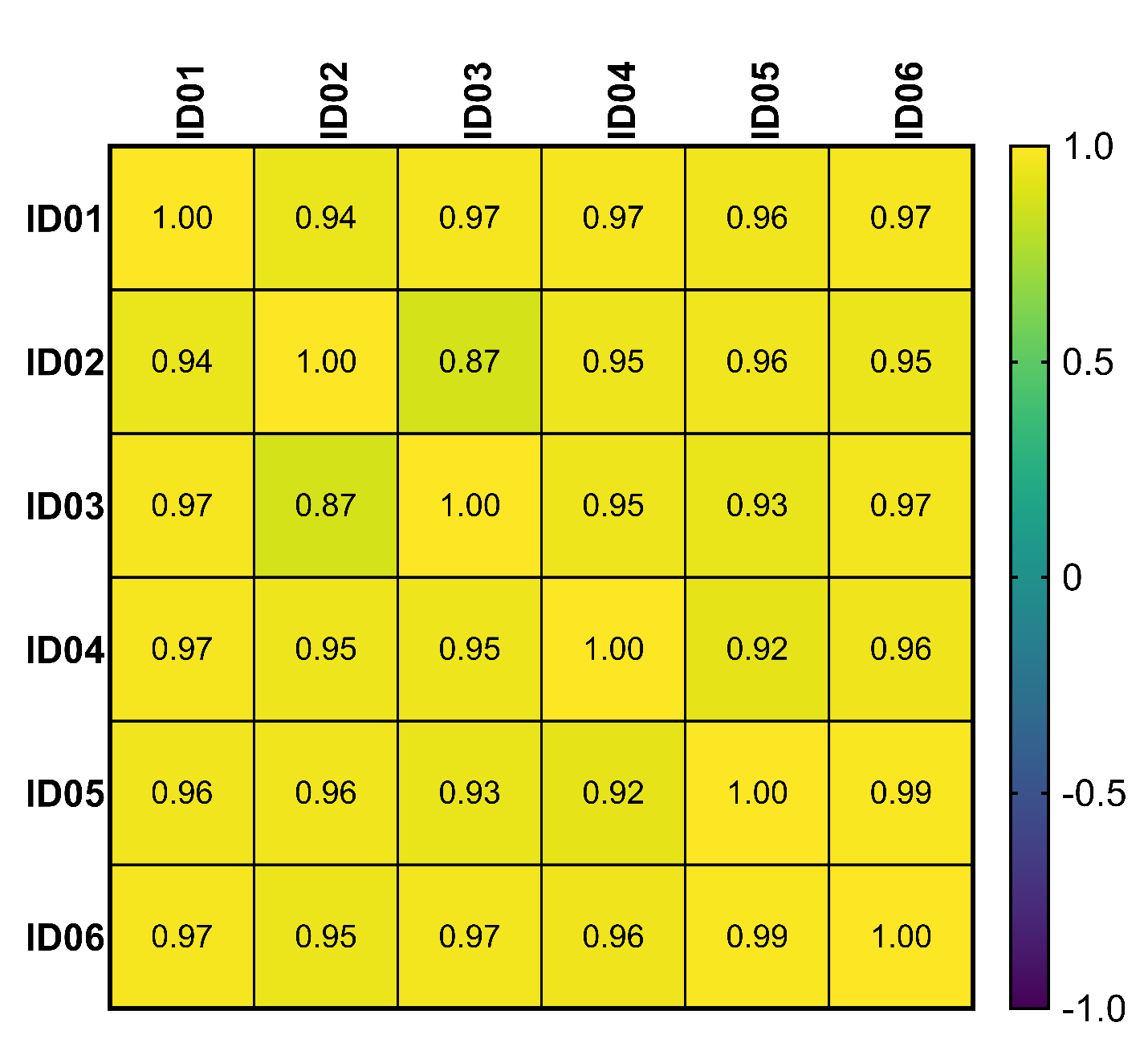

Supplement: Supplementary file 1 — Supplementary Material 1 [file 12149_2025_2144_MOESM1_ESM.docx]
